# Prevalence of problematic internet use disorder and associated risk factors and complications among Iranian university students: a national survey

**DOI:** 10.15171/hpp.2019.29

**Published:** 2019-08-06

**Authors:** Jalal Poorolajal, Jamal Ahmadpoor, Younes Mohammadi, Ali Reza Soltanian, Seyedeh Zahra Asghari, Ehsan Mazloumi

**Affiliations:** ^1^Department of Epidemiology, School of Public Health, Hamadan University of Medical Sciences, Hamadan, Iran; ^2^Research Center for Health Sciences, School of Public Health, Hamadan University of Medical Sciences, Hamadan, Iran; ^3^Modeling of Non-communicable Diseases Research Center, School of Public Health, Hamadan University of Medical Sciences, Hamadan, Iran; ^4^Social Determinants of Health Research Center, School of Public Health, Hamadan University of Medical Sciences, Hamadan, Iran; ^5^Department of Biostatistics, School of Public Health, Hamadan University of Medical Sciences, Hamadan, Iran; ^6^Department of English Language and Persian Literature, School of Medicine, Hamadan University of Medical Sciences, Hamadan, Iran

**Keywords:** Internet, Video games, Social media, Mental health, Students, Suicide

## Abstract

**Background:** Despite the growing epidemic of problematic Internet use (PIU), little information is available on PIU and related factors in Iran.

**Methods: ** This cross-sectional study was conducted on 4261 university students among 13universities throughout the country in 2017. The data collection tool included demographic characteristics, the status of using the Internet, social media, computer games, tobacco, alcohol, and illicit drugs, suicide ideation and attempt, and unprotected sex. PIU was measured using the15-item PIU questionnaire. The status of general health was evaluated using the 28-item general health questionnaire (GHQ) questionnaire. The simple and multiple logistic regression analysis were used to measure the crude and adjusted associations between various factors and PIU.

** Results: ** Of 4261 university students, 55.9% were female, 13.5% were smokers, 4.9% were drug abusers, 7.9% were alcohol abusers, 7.8% had unprotected sex in the past year, 7.4%had suicidal ideation in the past month, 1.7% had attempted suicide in the past year, and27.3% suffered from PIU. Only 61.1% had normal health. In contrast, 30.9%, 7.2%, and 0.8%had mild, moderate, and severe general health problems, respectively. There were significant relationships between PIU and age group 20-24 vs. <20 years (odds ratio [OR]=1.39; 95% CI:1.06, 1.82), single vs, married (OR=2.57; 95% CI: 1.85, 3.57), suicidal attempt (OR=2.77;95% CI: 1.47, 5.19), using online games (OR=1.31; 95% CI: 1.07, 1.60), and poor general health (OR=12.14; 95% CI: 4.53, 32.54).

**Conclusion:** Nearly one-third of medical sciences students suffered from PIU. This unhealthy behavior was associated with poor general health and elevated risk of suicidal behaviors. This health-threatening behavior provides an early warning signal that deserves special attention, otherwise, it may threaten both college students’ health and function.

## Introduction

The Internet is a very complex and revolutionary invention that has altered positively and negatively many aspects of real human life worldwide. Indeed, virtual space is not something apart from, but along real life. Most people use the Internet for activities such as information, education, business or entertainment.^1,2^ About 50% of the world’s Internet users live in Asian countries.^3^ Among countries in the Middle East, the number of Iranian social media and Internet users has increased substantially in the past decade. This figure has increased from 3.8% in 2000 to more than 68.5% in 2016.^4^

There were positive relationships between income, educational levels, the Internet and social media use. Those with higher levels of income and education are more likely to use the Internet and social media.^5^ The first reports about the overuse of the Internet released almost two decades ago.^6,7^ Since then, several studies were conducted on this subject, especially in the past decade.^8-10^

The problematic Internet use (PIU) is a consequence of the excessive use of the Internet. In general, PIU can be defined as “Use of the Internet that creates psychological, social, school, and/or work difficulties in a person’s life.”^11^ The results of eight studies carried out in the United States indicated that the prevalence of PIU among US college students ranged from 0% to 26.3%.^12^ Similar epidemiological studies showed that the prevalence of Internet addiction among Iranian university students ranged from 3.6% to 28.7%.^13-16^

Many studies have shown that inappropriate and excessive use of the Internet to be associated with a variety of psychosocial problems, such as anxiety,^17^ depression,^18^ insomnia,^19,20^ suicidal ideation,^21,22^ social phobia,^23^ drug abuse,^24^ problematic alcohol use,^25^ attention deficit hyperactivity disorder,^26^ problems with family interactions,^27^ increased loneliness,^28^ poor general health and emotional well-being^29,30^ and demographic and socio-psychological characteristics.^31^

Several questionnaires have been developed to assess the dependence on the Internet. The 15-item PIU questionnaire was developed by Caplan in 2002.^11,32^ This questionnaire evaluates the excessive use of the Internet from different psychological aspects, including a preference for online social interaction, mood regulation, cognitive preoccupation, compulsive internet use, and negative outcomes. Despite the growing epidemic of PIU among college students, little information is available on the predisposing factors and complications of the Internet overuse in the developing countries. Unless reliable information about the burden and associated risk factors of Internet addiction is collected, it is difficult or even impossible to design and carry out preventive measures. The aim of this study was to assess the prevalence of PIU and associated predisposing factors and complications among Iranian university students at the national level using the 15-item PIU questionnaires.

## Materials and Methods

### Design and participants

This cross-sectional study was carried out at 13 medical sciences universities throughout the country from October 2016 to December 2017. No special eligibility criteria were considered for enrolment. Both male and female university students from various degrees (bachelor, master, Ph.D., and MD) and disciplines (medicine, dentistry, pharmacology, nursing, midwifery, biochemistry, epidemiology, etc) were invited to participate in the study. The non-medical engineering universities were not included in this study just due to logistic problems. The students participated voluntarily and anonymously in the study. We emphasized on the first page of the questionnaire that participation in the study was entirely optional and they can return the blank questionnaire if they did not want to participate. The students were recruited into the study during their free time in the library, residence halls, coffee shops, or dormitories.

**Figure 1:**
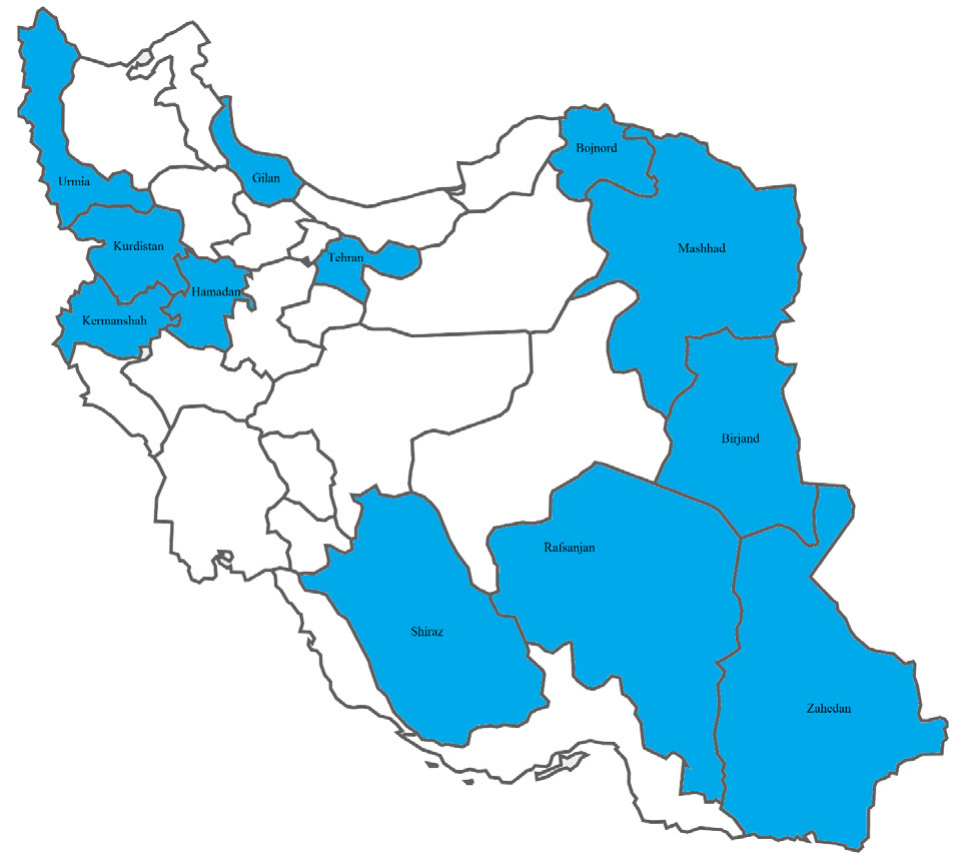
The provinces of Iran from where the samples were taken.

We took a sample of around 500 from big universities with more than 10 000 students, and a sample of around 250 from small ones with less than 10 000 students as follows: Kermanshah (492), Tehran (500), Shiraz (483), Mashhad (508), Hamadan (265), Kurdistan (247), Urmia (255), Gilan (251), Iran (250), Zahedan (251), Birjand (266), Bojnord (245), and Rafsanjan (248) (Figure 1). At each university, the samples were taken from both genders in different colleges and from various disciplines.

### Measures

The data collection tool was a self-administered questionnaire including demographic characteristics, duration and type of using the Internet, social media, and computer games, using tobacco, alcohol, and illicit drugs, suicide ideation and attempt, and unprotected sex. In addition, the Persian versions of the two standard questionnaires were given to the participants, including the 28-item general health questionnaire (GHQ-28)^33,34^ (Cronbach’s α reliability coefficient = 0.93) and the 15-item problematic internet use (PIU-15) questionnaire^11,32,35^ (Cronbach’s α reliability coefficient = 0.93).

### Problematic internet use

The PIU-15 questionnaire was used as a screening tool to evaluate five sub-scales, including (a) preference for online social interaction (items 1-3), (b) mood regulation (items 4-6), (c) cognitive preoccupation (items 7-9), (d) compulsive Internet use (items 10-12), and (e) negative outcomes (items 13-15). Every question had seven possible answers (on a scale of 1-7). The total score ranged from 15 to 105. Total scores of 15-59 were considered normal Internet use and 60-105 PIU.

### General health problems

The GHQ-28 questionnaire was used as a screening tool for measuring the general health, including somatic symptoms (items 1-7), anxiety/insomnia (items 8-14), social dysfunctions (items 15-21), and severe depression (items 22-28). Every question had four possible answers (on a scale of 0-4). The total score ranged from 0 to 84. Total scores of 0-22 were considered normal, 23-40 mild, 41-60 moderate, and 61-84 a severe general health problem.

### Statistical analysis

We used descriptive statistics for the analysis of categorical variables. Chi-square test was used for comparing categorical variables. The simple and multiple logistic regression models were used to measure the crude and adjusted association between various factors and PIU. The associations were reported as an odds ratio (OR­) with 95% confidence intervals (CI). All statistical analyses were performed at a significance level of 0.05 using Stata software version 14 (StataCorp, Texas, US).

## Results

The mean (SD) age of the participants was 22.17 (3.18) years, ranged from 18 to 46 years. Of the 4261 university students who participated in the study, 2379 (55.9%) were female, 575 (13.5%) were used tobacco in the past month, 210 (4.9%) were used illicit drugs in the past month, 334 (7.9%) were used alcoholic drinks in the past month, 332 (7.8%) had unprotected sex in the past year, 313 (7.4%) had suicidal ideation in the past month, 74 (1.7%) had attempted suicide in the past year, and 820 (19.3%) had a lack of goal clarity, 3827 (89.8%) were used the Internet for more than two hours a day, and 4073 (95.6%) were used the Internet at least one day a week.

According to the PIU-15 questionnaire, 3078 (72.7%) were normal Internet users, whereas 1151 (27.3%) were PIUs. Based on the GHQ-28 questionnaire, only 2598 (61.1%) students had normal general health. In contrast, 1314 (30.9%) had mild, 308 (7.2%) had moderate, and 33 (0.8%) had severe general health problems.

### Problematic internet use disorder and personal, behavioral and mental factors

The association between PIU and various factors is given in Table 1. Based on the unadjusted model, there were a significant association between PIU and some age groups, marital status, tobacco use, unprotected sex, suicidal ideation and attempt, lack of goal clarity, and the general health problems. Also, based on the adjusted model, there were a significant association between PIU and some age groups, marital status, alcohol use, and attempting suicide. According to these results, the odds ratio (95% CI) of PIU was associated with the age group of 20-24 year 1.39 (1.06, 1.82), being single 2.57 (1.85, 3.57), alcohol use 0.65 (0.43, 0.97), and suicide attempt 2.77 (1.47, 5.19). In addition, there was an increasing trend between the level of general health problems and PIU (*P*<0.001). The risk of PIU increased with the severity of the general health problems.

**Table 1:** The association between problematic Internet use (PIU) and various factors, using simple and multiple logistic regression models

**Variables**	**PIU**		**Model 1**		**Model 2**	
	**No**	**Yes**	**Unadjusted OR (95% CI)**	***P*** ** value**	**Adjusted OR (95% CI)** ^a^	***P*** ** value**
Gender						
Female	1728	636	1.00		1.00	
Male	1345	515	1.04 (0.91, 1.19)	0.538	1.00 (0.84, 1.18)	0.973
Age group (year)						
<20	407	119	1.00		1.00	
20-24	2019	844	1.43 (1.15, 1.78)	0.002	1.39 (1.06, 1.82)	0.014
25-29	404	125	1.05 (0.79, 1.41)	0.754	1.30 (0.87, 1.93)	0.188
≥30	124	26	0.72 (0.45, 1.15)	0.209	1.07 (0.55, 2.06)	0.831
Marital status						
Married	451	73	1.00		1.00	
Single	2595	1072	2.55 (1.97, 3.30)	0.001	2.57 (1.85, 3.57)	0.001
Divorced	27	6	1.37 (0.55, 3.44)	0.520	0.38 (0.07, 1.92)	0.243
Educational level						
Bachelor of science	1382	565	1.00		1.00	
Master of science	289	98	0.83 (0.65, 1.06)	0.165	0.82 (0.57, 1.19)	0.316
Professional doctorate	1297	461	0.87 (0.75, 1.00)	0.058	0.91 (0.76, 1.08)	0.318
PhD/Resident	82	21	0.63 (0.38, 1.02)	0.060	0.66 (0.28, 1.57)	0.352
Past semester final grades						
A (90-100)	130	48	1.00		1.00	
B (70-89)	1143	371	0.87 (0.62, 1.25)	0.472	0.83 (0.56, 1.22)	0.362
C (50-69)	1027	395	1.04 (0.73, 0.48)	0.820	0.90 (0.61, 1.32)	0.602
D (<49)	235	127	1.46 (0.98, 2.17)	0.059	1.29 (0.83, 2.00)	0.245
Tobacco use (past month)						
No	2689	963	1.00		1.00	
Yes	385	186	1.34 (1.11, 1.63)	0.002	1.03 (0.77, 1.37)	0.822
Drug abuse (past month)						
No	2929	1083	1.00		1.00	
Yes	143	65	1.22 (0.90, 1.66)	0.179	0.65 (0.41, 1.02)	0.066
Alcohol use (past month)						
No	2844	1053	1.00		1.00	
Yes	232	96	1.11 (0.87, 1.43)	0.380	0.65 (0.43, 0.97)	0.039
Unprotected sex (past year)						
No	2864	1030	1.00		1.00	
Yes	213	119	1.55 (1.22, 1.96)	0.001	1.26 (0.89, 1.78)	0.183
Suicide ideation (past month)						
No	2879	1034	1.00		1.00	
Yes	198	115	1.61 (1.27, 2.05)	0.001	0.72 (0.51, 1.04)	0.082
Suicide attempt (past year)						
No	3039	1112	1.00		1.00	
Yes	37	37	2.73 (1.72, 4.33)	0.001	2.77 (1.47, 5.19)	0.001
Lack of goal clarity						
No	2523	887	1.00		1.00	
Yes	554	262	1.34 (1.13, 1.58)	0.001	1.05 (0.86, 1.30)	0.589
General health						
Normal	2140	450	1.00		1.00	
Mild	776	722	3.19 (2.75, 3.71)	0.001	3.19 (2.68, 3.81)	0.001
Moderate	153	155	4.81 (3.76, 6.15)	0.001	4.89 (3.63, 6.60)	0.001
Severe	9	24	12.68 (5.85, 27.46)	0.001	12.14 (4.53, 32.54)	0.001

^a^ Adjusted for all variables in the table.

### Problematic internet use and online behaviors

The association between PIU and using the Internet, social media, and online games are given in Table 2. There was a dose-response relationship between PIU and the numbers hours using the Internet per day and per week. There was no significant association between PIU and using social media, but a significant relationship was observed between PIU and using online games, OR=1.31 (1.07, 1.60).

**Table 2:** The association between problematic Internet use (PIU) and using the Internet, social media, online games, and Internet addiction, and the general health, using simple and multiple logistic regression models

**Variables**	**PIU**		**Model 1**		**Model 2**	
	**No**	**Yes**	**Unadjusted OR (95% CI)**	***P*** ** value**	**Adjusted OR (95% CI)** ^a^	***P*** ** value**
Internet use (hour/day)						
<2:00	298	29	1.00		1.00	
2:00-3:59	1111	298	2.75 (1.84, 4.12)	0.001	2.88 (1.74, 4.78)	0.001
4:00-5:59	973	403	4.25 (2.85, 6.34)	0.001	3.86 (2.33, 6.38)	0.001
6:00-7:59	306	186	6.24 (4.09, 9.53)	0.001	5.53 (3.26, 9.38)	0.001
8:00-9:59	135	83	6.31 (3.95, 10.09)	0.001	6.55 (3.66, 11.71)	0.001
≥10:00	168	139	8.50 (5.46, 13.23)	0.001	6.85 (3.91, 11.99)	0.001
Internet use (day/week)	-	-	1.25 (1.15, 1.35)	0.001	1.34 (1.21, 1.48)	0.001
Using social media						
No	184	52	1.00		1.00	
Yes	2873	1097	1.35 (0.99, 1.85)	0.062	1.09 (0.73, 1.64)	0.649
Using online games						
No	2510	869	1.00		1.00	
Yes	542	278	1.48 (1.25, 1.74)	0.001	1.31 (1.07, 1.60)	0.008

^a^Adjusted for gender, age, marital status, educational level, past semester final grades, tobacco or drug or alcohol use, unprotected sex, suicide ideation or attempt, lack of goal clarity, and general health.

## Discussion

The prevalence of internet use disorder and related predisposing factors among Iranian university students was estimated by using PIU instruments. The results indicated that more than 27% of the students suffered from PIU. Several factors were associated with PIU including age, marital status, suicide attempts, poor general health, and using online games. Of course, the presence of association does not mean causation. However, the association between PIU and behavioral risk factors suggests that PIU may have common biological, psychological, social, environmental sources.

Several tools have been developed to address Internet addiction. But the 15-item PIU questionnaire, which was developed by Caplan^11,32^ evaluates the excessive use of the Internet from different psychological aspects. PIU means the use of the Internet that creates psychological, social, school, and/or work difficulties in a person’s life^11^ which is assessed by the PIU-15 questionnaire. This questionnaire has five sub-scales, including (a) preference for online social interaction; (b) mood regulation; (c) cognitive preoccupation; (d) compulsive internet use; and (e) negative outcomes. This means that the PIU-15 questionnaire makes a distinction between the cognitive and behavioral aspects of deficient self-regulation.^32^ However, recent investigations have produced empirical evidence suggesting that compulsive Internet use is a central component of PIU.^36-38^ Furthermore, deficient self-regulation represents a higher-order construct that explains the interaction between compulsive behavioral symptoms and obsessive cognitive symptoms.^32^ In addition, Caplan showed that mood regulation was an important cognitive predictor of negative outcomes related to PIU.^11^

Although overuse of the Internet and computer games may be harmful, using moderate video games may be pleasant and useful. A study, conducted by Allahverdipour et al in 2010,^39^ examined the relationship between video game playing and psychological well-being and aggressive behaviors. They concluded that moderate use of the Internet and video game playing may have a positive effect on the adolescents’ mental health, although excessive gamers showed mild increases in problematic behaviors.

Our results revealed a significant association between PIU and suicidal behaviors and poor general health. The relationship between PIU and suicidal behaviors was reported in the previous literature.^40-42^ Furthermore, the incidence of suicide increases with risky behaviors such as drug abuse,^43^ alcohol abuse,^44^ smoking,^45^ and unprotected sex.^22^ On the other hand, evidence has shown that PIU is associated with a wide variety of psychosocial problems, such as anxiety,^17^ depression,^18^ bipolar disorder,^42^ Loneliness,^28^ and poor general health.^29,30^ Indeed, PIU may result in psychological and emotional changes and provides a complex interplay of biological, psychological, and environmental factors that may promote suicidal behaviors.

Our findings indicated that the prevalence of poor general health, as well as high-risk behaviors, such as tobacco use, illicit drug abuse, alcohol abuse, and unprotected sex, were relatively high among university students. Previous studies conducted in different parts of Iran have reported similar results.^22,30,46-48^ Risky behaviors exhibited by some students can adversely affect their overall development and well-being as a youth.^49^ Evidence shows a negative association between high-risk behaviors and academic achievement and success. This means that students with higher grades are less likely to participate in high-risk behaviors than their classmates with lower grades, and students who do not participate in high-risk behaviors receive higher grades than their classmates who do participate in high-risk behaviors.^50^ Indeed, there is a negative interaction between risky behaviors and academic achievement. Therefore, there is an urgent need for implementing prevention and education programs, to reduce PIU and related risky behaviors among college students.

### Limitations

There are a few limitations involved in this study that must be addressed. Initially, this study, like any other cross-sectional study, had an inherent bias, because these types of studies measure the exposure and the outcome at the same time. Since cross-sectional studies have no dimension of time, they cannot support causal relationships. In addition, university students participated voluntarily in the study and filled out an anonymous self-administered questionnaire. Indeed, data were collected subjectively rather than objectively. Therefore, it is likely that they did not give the correct answer to some sensitive questions which are considered cultural taboos in our country such as unprotected sex, alcohol and drug abuse. Therefore, the prevalence of these factors might be underestimated. Furthermore, our data collection tool was rather bulky included more than 70 questions. This amount of questions seems boring and it is possible that some participants answered questions carelessly. Finally, the technical and engineering students were not enrolled in this study, therefore, the results of this study cannot be attributed to these types of students.

### Strengths

While these limitations are important, this cross-sectional study was conducted at the national level consisted of 13 medical universities involving 4261 students of various disciplines and educational levels. Therefore, the results of this study can reflect a good view of PIU and associated factors and complications among medical university students in a high-middle income country in the heart of the Middle East.

## Conclusion

The results of this study suggested that nearly one-third of medical sciences students suffered from PIU. Our findings indicated that students suffering from PIU were more likely to experience health-threatening conditions such as poor general health and increased risk of suicidal behaviors. These findings provide an early warning signal that deserves special attention, otherwise, may impair the students’ academic achievement and success and cause damage to the students’ function.

PIU is the consequence of Internet overuse. On the other hand, PIU is associated with psychological, social, carrier difficulties. Therefore, education and improving learners’ information about the malicious effects of the Internet overuse during school education and the International Computer Driving License (ICDL) class may be useful and effective.

## Ethical approval

This study was approved by the Ethics Committee of The Hamadan University of Medical Sciences (IR.UMSHA.REC.1395.433). All university students participated voluntarily in this study.

## Competing interests

The authors declare that they have no competing interests.

## Funding

The Vice-Chancellor of Research and Technology, Hamadan University of Medical Sciences funded this study (grant no. 9511126676). The Vice-Chancellor of Research and Technology, had no role in the study design, collection, analysis or interpretation of the data, writing the manuscript, or the decision to submit the paper for publication.

## Authors’ contributions

JP contributed to study conception and design, analysis and interpretation of data, and drafting of the manuscript. JA contributed to study design, acquisition of data, analysis, and interpretation of data, and critical revision. YM contributed to the study design and critical revision. ARS contributed to study design and critical revision. SZA contributed to study design and critical revision. EM contributed to acquisition of data and critical revision.

## Acknowledgments

This was part of the MSc thesis in Epidemiology. We would like to appreciate The Modeling of Non-communicable Diseases Research Center and the Vice-Chancellor for Research and Technology of the Hamadan University of Medical Sciences for approval of this work.
